# Habitat and movement selection processes of American lobster/jakej within a restricted bay in the Bras d’Or Lake/Pitu’paq, Nova Scotia, Canada

**DOI:** 10.1186/s40462-024-00486-6

**Published:** 2024-07-02

**Authors:** Shannon Landovskis, Megan Bailey, Sara Iverson, Skyler Jeddore, Robert J. Lennox, Caelin Murray, Fred Whoriskey

**Affiliations:** 1 Dalhousie University, 6299 South St, Halifax, NS B3H 4R2 Canada; 2 Ocean Tracking Network, 1355 Oxford St, Halifax, NS B3H 3Z1 Canada; 3 Unama’ki Institute of Natural Resources, 4102 Shore Rd, Eskasoni, NS B1W 1M4 Canada; 4 NORCE Norwegian Research Centre Laboratory for Freshwater Ecology and Inland Fisheries, Bergen, Norway

**Keywords:** American lobster, Bras d’Or Lake, Habitat use, Acoustic telemetry, Movement ecology

## Abstract

American lobster inhabit the unique, brackish Bras d’Or Lake system, although densities are low compared to areas with similar habitats in the Atlantic Ocean. Nevertheless, lobsters are an important part of local First Nation (Mi’kmaq) food and culture. We used acoustic telemetry and habitat mapping, combined with local Mi’kmaw knowledge, to document the movements and habitat use of adult lobsters within a section of the Lake. Movement patterns of acoustically tagged individual lobsters were analyzed with both resource selection functions and integrated step selection functions using data obtained from a high-resolution VEMCO Positioning System within a restricted bay in the Bras d’Or Lake. The resource selection function suggested stronger selections of substrates that contained a combination of soft and hard sediments. While the integrated step selection functions found substantial individual variability in habitat selections, there was a trend for lobsters to exhibit more resident behaviour on the combined soft/hard substrates despite the fact these sediments provided little in the way of obvious shelters for the animals. Adult lobsters at this site have very little risk of predation, which presumably allows them to freely exhibit exploratory behaviours and reduce their association with substrates that provide shelters.

## Introduction

**Fig. 1:**
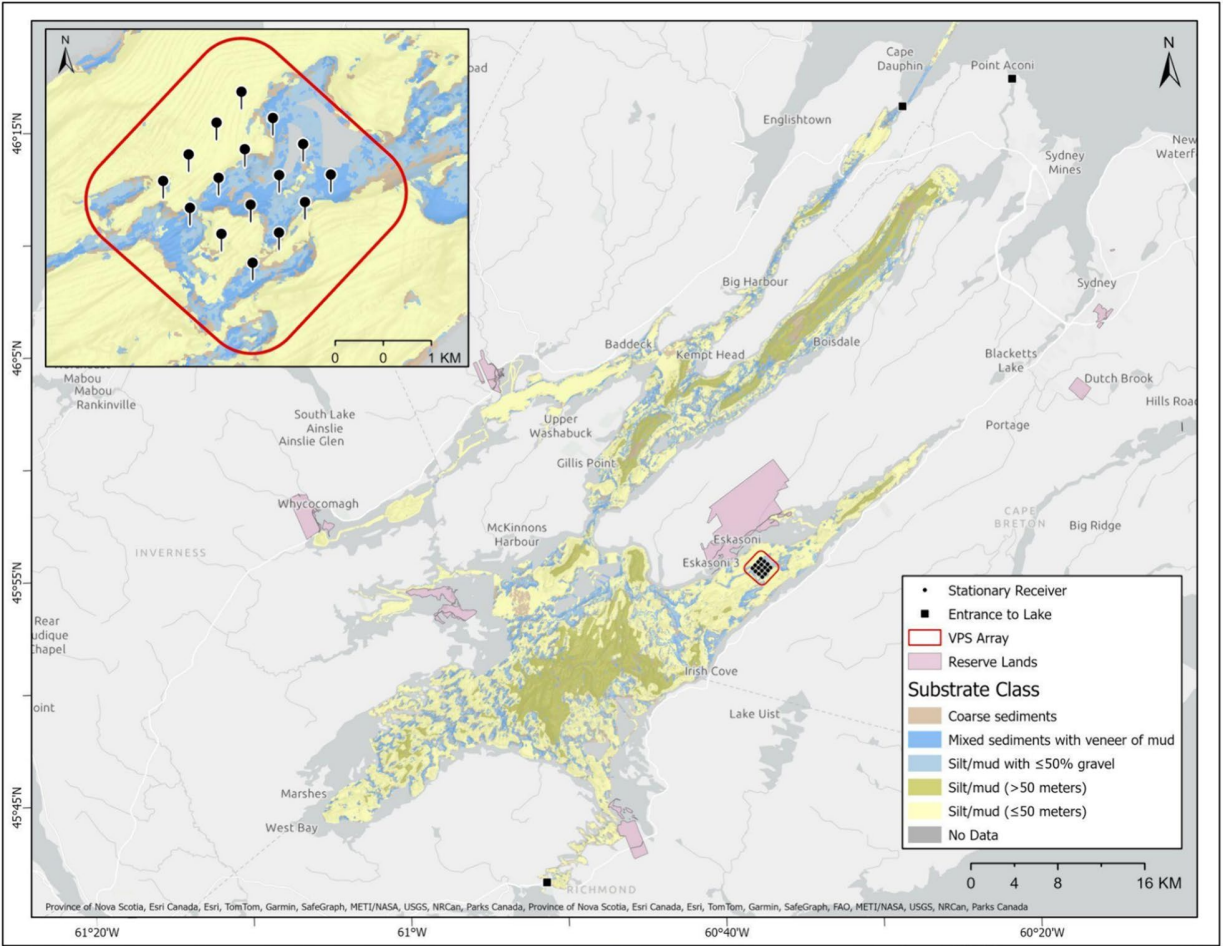
Map of the Bras d’Or Lake with substrate classes. Receiver locations (n = 16), within the East Bay, were deployed as a VEMCO Positioning System (VPS) and are outlined by the red diamond. The inset map shows a close up of the VPS array and the substrate in the area

The Bras d’Or Lake/Pitu’paq, Cape Breton/Unama’ki, Nova Scotia (Fig. 1) is a large (3600 km^2^) and unique estuarine environment in Mi’kma’ki, the ancestral home of the Mi’kmaq Indigenous people. It is comprised of two basins, multiple shallow and deep bays, and narrow channels and straits, with three outlets to the ocean and inputs from six rivers which results in salinity ranging from 20 to 26 ppt [28, 61]. The Lake has been designated as a UNESCO Biosphere Reserve and is populated by a diversity of marine and anadromous species [21, 28, 61]. These valued species are critical to the food security and culture of the Mi’kmaq, with the American lobster (Mi’kmaw: jakej, *Homarus americanus*) being a particularly important native species. Lobster densities within the Bras d’Or Lake are low compared to areas with similar habitats immediately outside of the Lake along the Atlantic coast [43, 63] and Mi’kmaw ecological knowledge indicates that these densities are lower now than within recent memory (S. Denny, pers. comm.). Low egg production, poor food quantity or quality, limited habitat, and hyposalinity are commonly cited as potential factors that limit lobster production [43], however, studies have not yet confirmed the impact of any of these factors on lobster populations in the Bras d’Or Lake [61]. It is thought that there is the potential to increase lobster abundance by addressing constraints to egg production and habitat availability [43, 63], but egg production in the Lake is only considered to be limited by the density of lobsters. It has been suggested that there is potential for habitat enhancement to increase lobster abundances, however, at present it is not known how much suitable habitat for lobsters there is in the Lake, how lobsters use the currently available habitat in the system, and what lobsters in the Lake experience as “quality habitat” [14, 61, 63].

Substrate type influences lobster movement characteristics, such as movement step lengths and turning angles. Low structure and soft substrates favour high speed and high directionality movements [55], whereas medium–high structure combined with hard substrates necessitate or promote slower speeds and lower directionality [47, 55, 72]. Behaviour can also be inferred from movement characteristics, with shorter step lengths and lower directionality indicating searching or foraging movements, while longer step lengths and higher directionality indicate exploratory movements [36, 55]. Benthic substrates in the Lake had previously been surveyed with multi-beam sonar [50, 51, 61, 65], but until very recently the data had not been processed to identify and quantify the habitat types present [41]. The majority of the bottom appears to be sandy-to-silty substrate (low structure), which is generally considered suboptimal for lobsters as it does not provide shelter from predators and may be food-poor (but see [6], https://www.youtube.com/watch?v=yuE7XeqNLl0). Within the Lake, there are areas with boulder and cobble (medium and high structure) that may be more suitable for most post-larval stages of the species [43].

Given the importance of substrate to lobsters, a systematic and quantitative evaluation of the available substrate types in the Bras d’Or Lake and their link to local lobster biology is highly desirable. Knowledge about habitat is critical due to its role in explaining the local distribution and composition of lobster communities, as well as its influence on lobster behaviour at various points in the species life cycle [6, 27, 55]. First Nations and the Government of Canada are working towards a path for evidence-based lobster management in the changing fishery of the Atlantic coast region, including the Bras d’Or Lake, a path that recognizes and affirms Mi’kmaw Treaty and constitutional rights to fish and incorporates Mi’kmaq knowledge. With a Treaty fishery comes the potential for increased lobster fishing in areas of the Lake, like East Bay, that have previously been unexploited by commercial harvesters [43, 61, 62]. Given the potential for increased fishing pressure in the area, western and Mi’kmaw knowledge holders are coming together to identify shared gaps in knowledge that could inform fishery and conservation planning, and to co-develop research programs to address them.

This study was developed out of that need for increased collaboration, specifically through a collaborative project called Apoqnmatulti’k (Mi’kmaw for “we help each other”). Apoqnmatulti’k is addressing some of these persistent knowledge gaps, and doing so in a way that respects different knowledge paradigms. This project was a space for Mi’kmaw and western knowledge holders to work together and share ways of knowing, thus adding depth to both the research and the relationships amongst project partners. As part of Apoqnmatulti’k, the research plan, study design, tagging methodologies, and research questions were co-developed to ensure they aligned with partner knowledge systems and upheld the values of all involved. Benthoscape mapping, which models substrate and geomorphology, was completed for the entire Bras d’Or Lake to address a shared knowledge gap [41]. This new knowledge of the habitat is combined here with habitat-selection analyses derived from movement and residence patterns of acoustically tagged American lobsters to provide insights into the species’ habitat selection processes in this unique ecosystem. Resource selection functions (RSF) are weighting functions to describe the probability that animals are located in a specific habitat over alternatives [37]. RSF typically use observation data rather than movement data. Alternatively, integrated step-selection functions (iSSF) are a weighting function to test the probability of selecting a given habitat against possible alternatives that were available to the individual [37, 53]. RSF and iSSF have rarely been applied to aquatic telemetry data, therefore, a key objective of this work was to combine these analytical methods to test which substrate classes lobster used in the low productivity estuarine bay study area. This paper addresses two key questions: (1) What specific substrates do lobsters select in this unique area? and (2) Are lobster movements and site fidelity altered based on the substrate type they are occupying?

## Methods

### Researcher positionality

Apoqnmatulti’k is a collaborative study that joins together those with different worldviews to conduct research in an environment built on equity and co-learning [20, 25]. Critical to this work was ensuring that all partners understood the knowledge and worldview that they were bringing to the group. As such, we offer a positionality statement here to support the reader in understanding the methods undertaken and our interpretation of the results. The work was completed during our journey together as non-Indigenous and Indigenous researchers through Apoqnmatulti’k. Throughout this process, we have strived to learn from one another and to continually question the influence that colonization has on our own practices and beliefs. This work is the result of many conversations held and decisions made among project partners as well as conversations with Mi’kmaw individuals from Eskasoni, the community where the work took place. Mi’kmaq is the plural non-possessive form of the word, while Mi’kmaw is the singular form, thus both will be used throughout.

### Study site and acoustic array design

This study took place within the East Bay/Tewitnu’jk of the Bras d’Or Lake/Pitu’paq (45.888890°, − 60.648236°; Fig. 1). The East Bay is located within Canada’s Department of Fisheries and Oceans (DFO) Lobster Fishing Area 28 (LFA 28), on the eastern side of the Lake. Acoustic telemetry was used to follow the movements of tagged lobsters. Sixteen acoustic receivers (InnovaSea VR2ARs) were deployed by staff of the Ocean Tracking Network (OTN) field team and the Unama’ki Institute of Natural Resources (UINR) from June 2019 until July 2021. While LFA 28 generally has low densities of lobster, receivers were placed within the centre of the Bay based on local Mi’kmaw knowledge of higher lobster densities than in other parts of the bay. These receivers were arranged into a gridded design (Fig. 1) to calculate animal positions based on time of arrival of a given tag’s signals on multiple receiver units using the VEMCO Positioning System (VPS). Positioning arrays increase the ability to assess habitat-use and small-scale movements within the grid by providing positions based on multilateralization [56]. The grid covered an area of 1395 m^2^ and testing provided a median position error within the array of 2.4 m.

### Lobster capture and tagging

Lobsters were captured and tagged between October and December 2019 (n = 4 female, 13 male) and again in December 2020 (n = 1 male), with the assistance of the project’s Community Liaison staff member and a local harvester from Eskasoni. Each lobster was tagged with a unique acoustic transmitter (model V13-1H-069 kHz, Vemco Division, InnovaSea Systems). The tags were 13 mm in diameter and transmitted a unique acoustic signal every 120–240 s, with an estimated battery life of 838 days. All tagged lobsters weighed more than 660g (tags were less than 2% of their body weight which evidence suggests does not alter the natural behaviour of lobsters [2, 24, 48]). Before tagging, the shell condition of each lobster was checked to confirm that each individual’s shells were fully hardened and showed no signs of moulting. Berried females were excluded from this study as the handling of berried females can contribute to losses of up to 50% of the brooded egg mass [64]. Lobster production in the Lake is low [43], thus UINR partners required that we avoided handling any berried females to remain aligned with study values and mitigate the impact this study could have on the lobster population. This study’s protocol was reviewed and approved by the Dalhousie Animal Care Committee (protocol I19-17).

### Habitat quantification

**Fig. 2:**
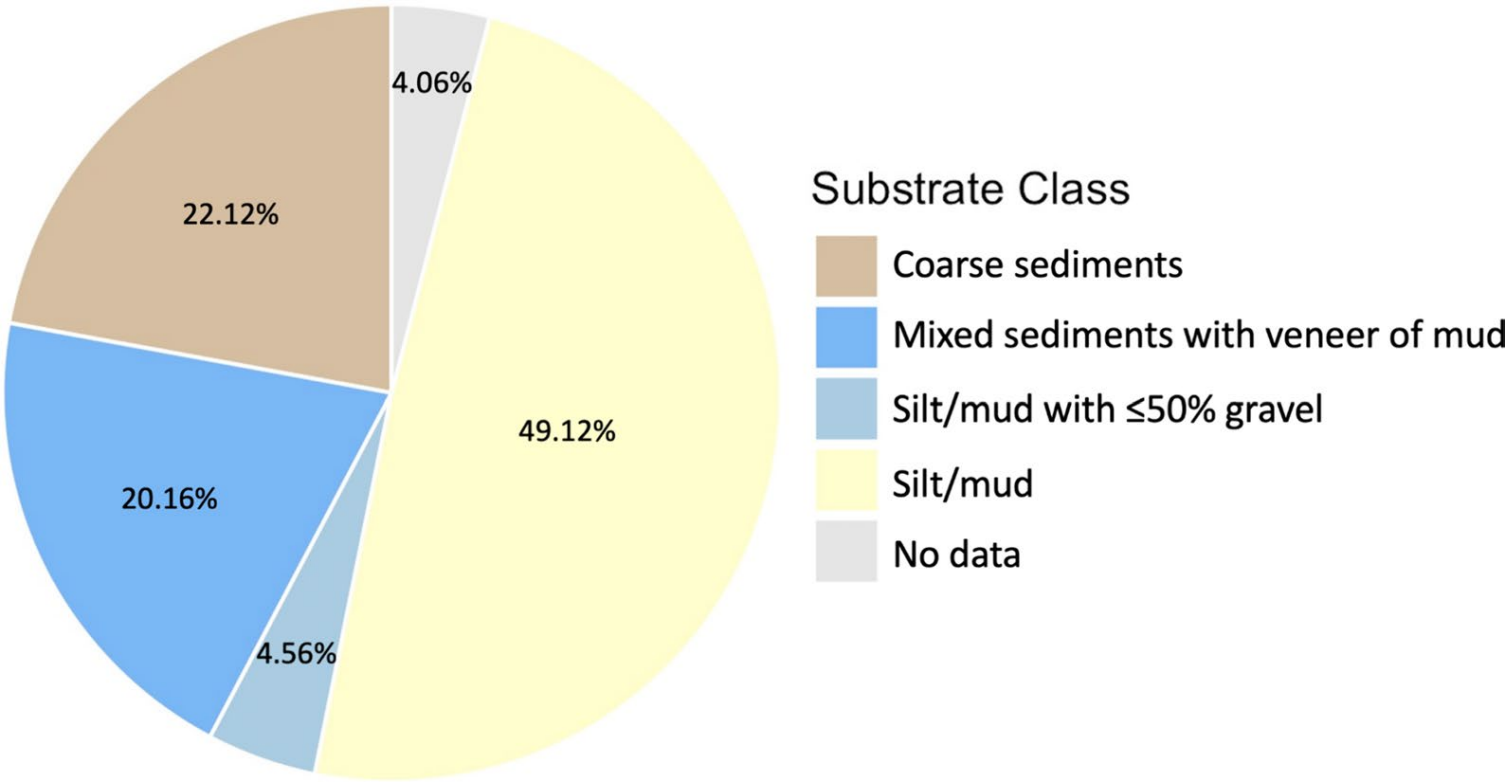
Relative availability of each substrate class found within the VPS array. Percent coverage of each substrate type within the VPS array is shown on each slice

Benthoscape mapping was completed using backscatter and bathymetry data from the Bras d’Or Lake collected through multibeam echosounder surveys conducted by the Canadian Geological Survey and the Canadian Hydrographic Service between 1999 and 2009. These surveys covered approximately 777.6 km^2^ of the lake, leaving some primarily inshore areas with no multibeam coverage [41, 50]. Several environmental variable layers were generated by Murray [41] from bathymetry and backscatter data and these variables were used to run a principal component analysis (PCA) to generate an input raster that would account for at least 95% of the variance between environmental covariate layers. An unsupervised Iso Cluster analysis was performed on the PCA input raster to group the data into discrete benthoscape classes. This Iso Cluster analysis was driven by an object-based image analysis segmentation method which was applied to the bathymetry and backscatter data with a spatial detail of 20, a spectral detail of 10, and a pixel size of 10 m in efforts to capture heterogeneity between classes in multidimensional space. This work was completed using ArcGIS Pro v. 2.8. The final benthoscape classification was developed and validated through ground-truthing with images from Shaw et al. [51] and newly collected video footage during this project using remotely operated vehicles (ROVs). The images were classified, using the Wentworth scale [68] and the Folk [15] method, into 5 substrate classes from 449 images: (1) *coarse sediments*, (2) *mixed sediments with a veneer of mud*, (3) *silt/mud with* ≤ *50% gravel*, (4) *silt/mud (at depths of* > *50 m)*, and (5) *silt/mud (at depths of less than* < *50 m)* (Table 1, [41], Fig. 2).

**Table 1:** Names and descriptions of the five substrate classes identified within the Bras d’Or Lake

Class	Name	Description
0	Coarse sediments	Mostly hard bottom with coarse fine sediments and no veneer of mud
1	Silt/mud with ≤ 50% gravel	A mixture of soft and hard bottom, such as gravel, cobble, and pebble
2	Mixed sediments with a veneer of mud	A mixture of soft and hard bottom with a veneer of mud and no presence of course, fine sediments
3	Deep Silt/Mud (> 50 m)	Soft bottom with no evidence of hard substrate, greater than 50 m depth
4	Shallow Silt/Mud (≤ 50 m)	Soft bottom with no evidence of hard substrate, less than or equal to 50 m depth

Murray [41]

### Telemetry data analysis

#### Data filtering

Raw detection data were offloaded periodically (at least once per year) from the deployed receivers. Data from within the VPS array were processed by InnovaSea and positions were calculated from their proprietary VPS software. Position data were then imported into R [45] for subsequent analysis. The horizontal positioning error (HPE) provided for each position calculation required filtering to eliminate suspicious positions. To enable this, a cut-off value was determined using detections of synchronization (sync) tags (n = 16) co-deployed at known positions within the VPS array (see [38]). HPE is an estimate of error sensitivity and is used to assess the position quality. It is unitless and relative, so HPE values are not comparable among studies [57]. A higher HPE value signifies that a position is of lower relative quality and is likely providing less reliable information about the position of the animal [57]. HPE cut-off determinations are made using the relationship between HPE and HPEm, which is the error in absolute terms and is provided by the VPS [8]. Each deployment (2019–2020, 2020–2021) of the receiver array was treated as an individual dataset and potential HPE cut-off values were determined for each by aggregating the HPE values and recording the lowest HPE that retained 95% of the original positions as a potential cut-off value.

Each yearly dataset was then filtered at each of the potential HPE cut-off values and the percent of original positions retained at that cut-off, as well as the median, 90th, and 95th percentiles of HPEm were calculated. The potential cut-off values were used to filter the lobster tag data for each dataset and then the percent of original positions retained was calculated. The final selected HPE cut-off value, HPE < 25, was chosen as a balance between having higher confidence in animal positions with the loss of spatial information resulting from the filtering and reducing sample size [8]. When the two datasets were combined and filtered at HPE < 25, the median HPEm for sync tags was 2.4 m and the 90th and 95th percentiles were 8.4 m and 11.9 m respectively. The ‘true’ position of animal tags cannot be known, thus HPEm values cannot be calculated. However, HPE calculations are conducted the same way for sync and animal tags so the absolute accuracy (HPEm) of the animal tags is expected to be similar to that of the sync tags whose true position is known [8]. To account for possible tagging effects on animal behaviour, any detections within 48 h of release were removed from the analysis (e.g., [3, 60], using tools in [71]).

#### Data preparation, RSF analysis, and iSSF analysis

Data were formatted according to the standards developed for analysis with the *amt* R package and analysed using both RSF and iSSF paired with the benthoscape map of the area [13, 41, 52, 53]. A sampling rate of 10 min was selected for the data (using *amt* [52, 53], and *dplyr* [71]). Both RSF and iSSF analyses extract the substrate where a position was recorded as the fixed effect in a binary regression, simple logistic regression for resource selection analysis and conditional logistic regression for iSSF. Benthoscape maps of the Bras d’Or from Murray (2021) were used and the *extract_covariates* function in *amt* was implemented to assign a habitat value to each position in the time series.

RSF analysis is a logistic regression of presences and pseudo-absences against covariates such as the habitat assigned to a point; the point being either a presence, which is a known position of an individual, or a pseudo-absence, which is a position available to the same individual, allows the regression model to estimate the probability of a point with a given habitat being selected or not [19]. In this analysis, the resource selection function therefore provided parameter estimates for the selection of four substrate types compared to the intercept value, *silt/mud with* ≤ *50% gravel*. This intercept value, which will be used to establish relative selection of other substrates, was selected as it was the least abundant substrate class within the array and used by all five individuals. For the RSF analysis, 10 pseudo-absences were added to the dataset using the *random_points* function in *amt* for each observed point, and the substrate at the presence and pseudo-absence points were extracted in *amt*. The RSF analysis was fitted using binary logistic regression in *mgcv* [73]. The model fitted whether a point was a presence or pseudo-absence as a function of the habitat type, with individual lobster considered as a random intercept. To limit the potential confounding effects of spatial autocorrelation that might affect the estimation of selection strength [13], the model was rerun 100 times with subsets of 100 presence and pseudo-absence points per individual. The random subsetting and rerunning produced 100 estimates of model coefficients that, when exponentiated, provided a relative selection strength for each substrate compared to the baseline level (*silt/mud with* ≤ *50% gravel*).

$${\text{gam}}({\text{true presence}}\,\sim \,{\text{substrate}} + s({\text{ID}},{\text{ bs}} = {\text{``re'' }})$$

iSSF analysis, a conditional logistic regression, provides a stronger inference than RSF analyses because it allows both movement and habitat-selection processes to be modelled simultaneously, thus exploiting the tracking data to explicitly model the movement decisions made by the animal [13]. However, in this study, there were only a few lobster tracks with sufficient data for iSSF analysis, so it was paired with the RSF analysis to facilitate a similar test of habitat selection while retaining more positions from the dataset. For the iSSF analysis, true steps were created, which are straight lines connecting two consecutive known animal positions, and then random steps were generated with the *random_steps* function, which uses a habitat-selection kernel multiplied by a selection-free movement kernel to determine an availability domain around each observed position and samples the specified number of random locations from within that domain, i.e. where the animal could have moved but did not at each step [13]. One hundred steps were selected for this analysis to optimize performance against potential bias, as a higher number of steps increases the computational burden and a lower number increases the estimation error [13, 53]. The substrate class was then extracted from both the beginning and the end of each step [52]. Any infinite log step length values were filtered out, along with any non-computable turning angles [71]. In iSSF analysis, a step length is the distance between the starting position and the ending position that were used to create each individual step. Bootstrap replicates (n = 1000) were generated using random sampling with replacement [70]. The conditional logistic regression model fitted for the iSSF by amt was:

$$\begin{aligned} & {\text{fit}}\_{\text{issf}}({\text{true step}}\,\sim \,{\text{ending substrate}}\, + \,{\text{log of step length}}\, \\ & \quad + \,{\text{cosine of turning angle}}\, + \,{\text{starting substrate}}:{\text{log of step length}}\, \\ & \quad + \,{\text{starting substrate}}:{\text{cosine of turning angle}}\, + \,{\text{stratified steps}}) \\ \end{aligned}$$

The first predictor variable was used to infer habitat selection processes, while the two predictor variables with interactions were used to infer movement processes [13, 53]. Interactions are important to step selection functions to account for potential nonlinearity between multiple predictors, particularly the substratum and the movement processes (turning and step length [13]). The final predictor variable accounted for the stratified steps, which include the observed step and the random steps associated with it [53]. The code required to complete the above is provided on Github (https://github.com/slandovs/bdl-lobster/tree/main).

To infer movement processes, biological interpretations were applied to the means of the bootstrapped estimates from the models that were iteratively re-fitted. For interactions between the substrate and the cosine of the turning angle, when the mean estimate was negative it suggested more turning and less directed movement on that substratum. For interactions between the substrate and the log of the step length, when the mean estimate was negative it indicated that lobsters were spending more time on that substratum because they were moving slower. When the mean estimate was positive, it indicated a gamma distribution shape parameter more concentrated away from 0 than if the individual had been on the reference class and was interpreted as an individual exhibiting longer step lengths [53, 54].

## Results

### Habitat quantification

The overall accuracy of the Lake benthoscape was 62.7% with a kappa statistic of 0.57%. A kappa value of 0.40–0.80 indicates moderate agreement [29]. The *silt/mud* class was the most accurately classified substrate with a user’s accuracy of (73.4%) followed by *mixed sediments* (50%), *silt/mud with* ≤ *50% gravel* (33.3%), and *coarse sediments* (12%). *Mixed sediments* were occasionally confused with *coarse sediments*, as expected, as both classes occurred in areas of medium to high backscatter returns and each class had limited ground-truthing images available for validation.

### Telemetry analysis

Hyperbolic positioning using time difference of arrival with the VPS yielded a total of 41,999 animal positions, reduced to 38,527 after the filtering protocol to remove unlikely positions. Of the 18 lobsters tagged for this study, five were never detected within this array, thus the 38,527 positions were attributed to 13 individuals, though three of these individuals had less than 20 positions and were removed from the data set before final analysis.

#### RSF analysis

**Fig. 3:**
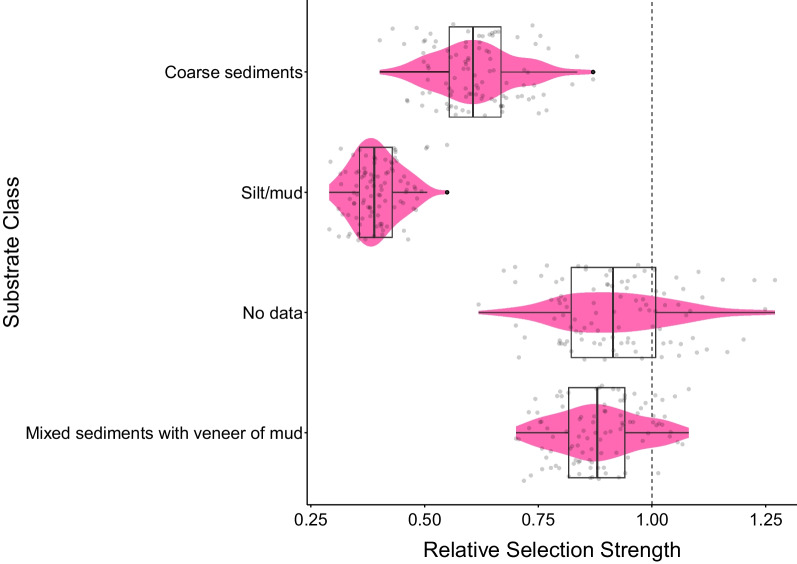
The relative selection strength of different substrate classes by ten lobsters using resource selection analysis. The exponentiated coefficients for each substrate are compared to the baseline level (*silt/mud with* ≤ *50% gravel)*. Data to the left of the dashed line (1.0) indicates reduced odds of selecting a substrate type, while data to the right of the dashed line indicates increased odds of selection. Boxes cover the interquartile range with the centre line at the median

**Fig. 4:**
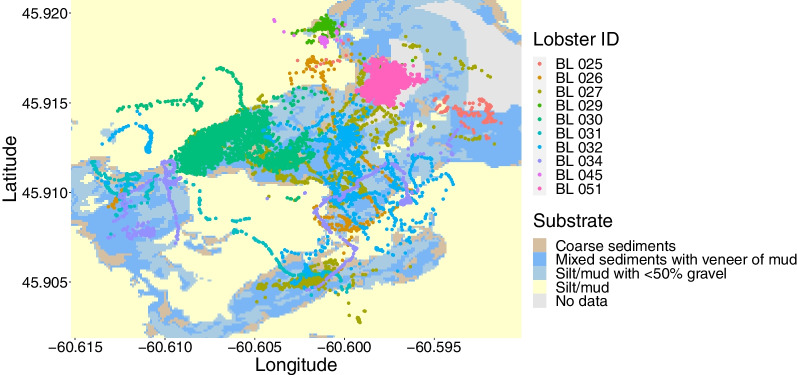
Positions of ten lobsters within the study focal area in relation to the different substrates available within the East Bay/Tewitnu’jk in the Bras d’Or Lake/Pitu’paq. Lobster positions were obtained through fine-scale positioning using a VEMCO Positioning System (VPS) array (n = 16 receivers)

Data from ten individuals provided evidence that lobsters selected *silt/mud with* ≤ *50% gravel* (intercept) more frequently than *coarse sediments* and *silt/mud*, despite *coarse sediment* representing 22.12% of the available substrate and *silt/mud* representing 49.12% of the available substrate within the VPS array (Figs. 2 and 3). The odds of a lobster selecting *silt/mud*, the dominant available substrate, were lowest, while *mixed sediments with a veneer of mud* and the area that had no substrate data had both negative and positive effects on selection, potentially indicating no selection (Figs. 2 and 3). Positions of these lobsters are shown in Fig. 4.

#### iSSF analysis

**Fig. 5:**
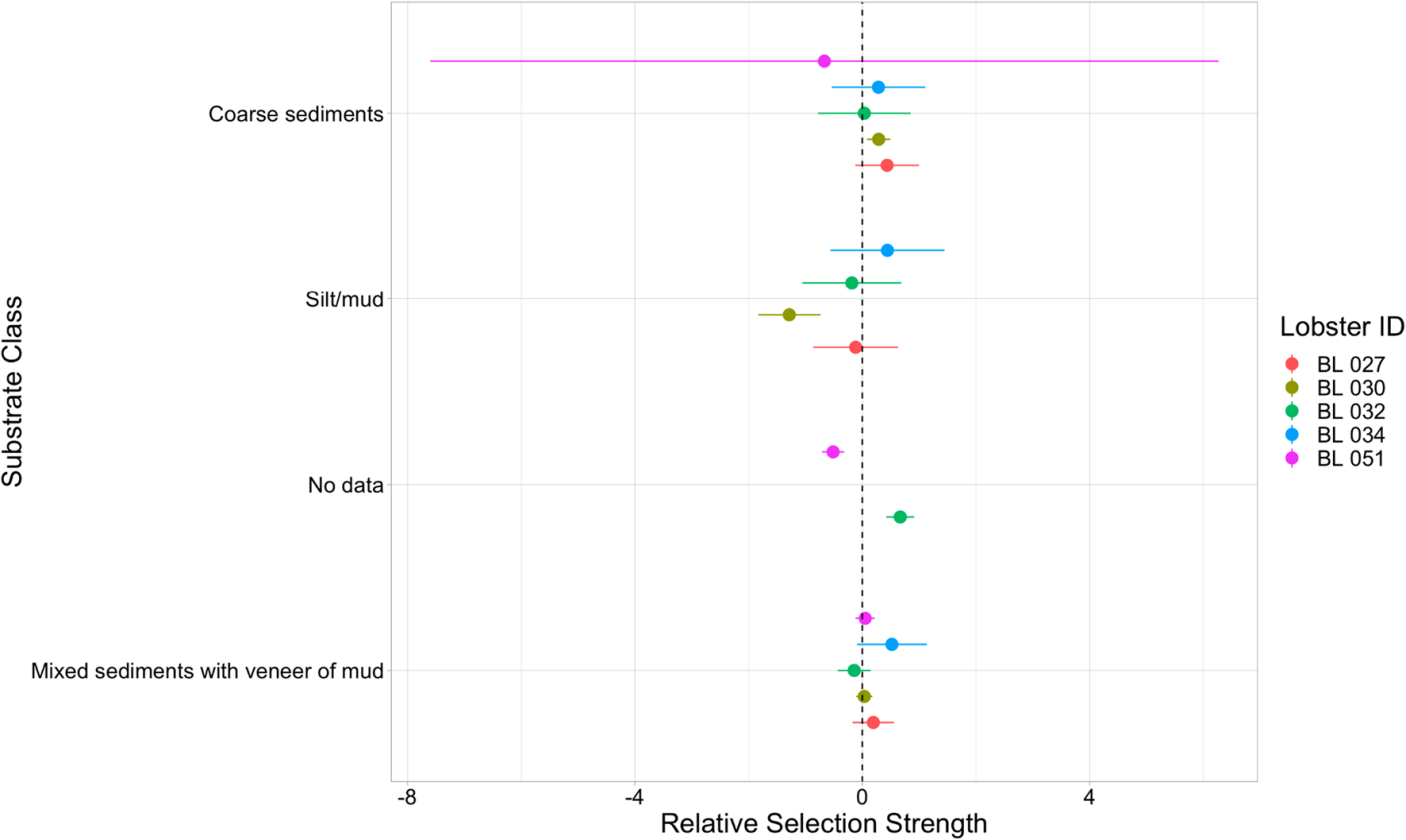
Relative selection strength of different substrate classes by five lobsters using point estimates (points) with 95% confidence intervals (horizontal lines) [1, 53]. No preference relative to the reference class (*silt/mud with* ≤ *50% gravel)* was indicated by intervals crossing the dashed vertical line. Negative values indicate less use of a habitat type than the reference class and positive values indicate more use of the habitat type than the reference class

Data from five individuals met the iSSF analysis criteria and were used in the analysis. No strong selection patterns were identified, with individual variability in selections of substratum compared to the baseline, *silt/mud with* ≤ *50% gravel* (Fig. 5). Animals BL 030, BL 032, and BL 034 had longer steps with higher directionality on both *mixed sediments with a veneer of mud* and *silt/mud* (Tables 2 and 3), indicating more exploratory movements on these substrates. Steps taken by lobsters tended to be significantly shorter on *silt/mud with* ≤ *50% gravel* than steps taken on other substrates (Table 3), indicating more resident behaviour on this substrate. BL 030 had greater odds of ending a step on the reference substrate than on *silt/mud*, but had greater odds of ending on *coarse sediment*. BL 032 had the greatest odds of moving onto areas with no data, BL 051 appeared to have lower odds (0.60) of moving onto areas with no data than *silt/mud with* ≤ *50% gravel* (Fig. 5). Collectively, the iSSF analyses exhibited how lobsters had relatively individualistic step selection on the substrates that were studied.

**Table 2:** Turning angle model outputs of individual lobster from the bootstrapped fitted integrated step selection functions

Term	BL 027		BL 030		BL 032		BL 034		BL 051	
	Estimate	SD	Estimate	SD	Estimate	SD	Estimate	SD	Estimate	SD
Mixed sediment	− 0.49	0.16	0.27	0.08	0.45	0.13	1.21	0.42	− 0.11	0.35
Silt/mud	− 0.86	0.46	0.32	0.22	1.60	0.40	0.61	0.40	–	–
Coarse sediment	− 0.33	0.31	− 0.14	0.11	2.01	0.79	1.28	1.37	2.16	28.43
No data	–	–	–	–	− 0.24	0.12	–	–	− 0.19	0.33

The estimate is the mean estimate from the bootstrapped data and the standard deviation (SD) is the standard deviation of the estimate within the bootstrapped data. All values are relative to the reference substrate, *silt/mud with* ≤ *50% gravel*. Negative values are interpreted as lower directionality on the respective substrate when compared to the reference substrate and positive values are interpreted as higher directionality

**Table 3:** Step length model outputs of individual lobster from the bootstrapped fitted integrated step selection functions

Term	BL 027		BL 030		BL 032		BL 034		BL 051	
	Estimate	SD	Estimate	SD	Estimate	SD	Estimate	SD	Estimate	SD
Mixed sediment	− 0.08	0.06	0.37	0.05	0.22	0.06	0.49	0.11	0.03	0.05
Silt/mud	0.13	0.18	0.79	0.17	1.51	0.28	0.30	0.13	–	–
Coarse sediment	0.06	0.13	0.21	0.07	3.12	0.87	0.43	0.21	4.14	8.87
No data	–	–	–	–	0.08	0.03	–	–	0.09	0.04

The estimate is the mean estimate from the bootstrapped data and the standard deviation (SD) is the standard deviation of the estimate within the bootstrapped data. All values are relative to the reference substrate, *silt/mud with* ≤ *50% gravel*. Negative values are interpreted as shorter step lengths on the respective substrate when compared to the reference substrate and positive values are interpreted as longer step lengths

## Discussion

While data usable for iSSFs were procured from only five of the 18 tagged individuals, the RSF analyses were able to be conducted on 10 tagged individuals. Despite these limited sample sizes, the positions provided by the acoustic telemetry system were considered to have high precision, which uniquely provides the possibility to conduct high dimensional analyses of habitat selection similar to those that have been developed in terrestrial environments [53, 54]. There were not consistent strong associations with a specific habitat in the iSSF analysis, but RSF habitat analysis revealed a trend towards stronger selection by lobsters for the *silt/mud with* ≤ *50% gravel*, which was not the most abundant habitat type (*silt/mud*, Fig. 2) at the site. The preference shown for this substrate is unlikely to be shelter related, as lobsters prefer areas with boulders for sheltering. Thus this preference may be related to foraging [17, 22, 49, 74].

These results are, to our knowledge, the first use of iSSFs to describe habitat and movement selection processes of an aquatic species in their natural habitat using acoustic telemetry. Deriving accurate positions for many individuals in an open marine environment is extremely challenging because there are few tools available to obtain reliable positions over extended time periods. Light-based geolocation tags have high position error and gridded acoustic receiver arrays for position calculations require many costly and closely spaced units to allow multilateralization, which prohibits their use in most instances [33]. The relatively small number of animals for which we were able to derive precise positions, and prolonged tracks for iSSF analysis, reflects these challenges and contextualizes the resource needs necessary for understanding marine species’ movements and habitat requirements at fine scales.

Bras d’Or Lake lobsters can exhibit high fidelity to the East Bay, suggesting that the local lobster population may be isolated from those in other parts of Bras d’Or Lake and from the broader Atlantic Ocean ecosystem (S. Denny, pers. comm.). The complex, multi-basin geography of the Lake, whereby narrow channels connect the basins to each other and the Lake to the Atlantic Ocean, is presumed to maintain this isolation by limiting both exchange of planktonic larvae as well as movements by juveniles and adults among habitats. Within the Atlantic Ocean, American lobster populations demonstrate a mosaic of generally predictable movement patterns and varying degrees of site fidelity. Seasonally, the majority of the individuals in many populations will move from shallow inshore feeding areas to deeper, offshore winter refuges, especially where inshore shelter is limited [3, 4, 18, 40]. However, populations will have individuals that may not undertake this seasonal movement [3], and the Bras d’Or Lake lobsters we tagged clearly did not leave the Lake for the Atlantic Ocean to overwinter. Consistent with this finding, all acoustically tracked berried female lobsters tagged in a New Hampshire estuary in autumn were within 1 km of their fall positions the following May–June [39]. In contrast, other researchers have found that ovigerous females may move from shallow to deeper water seeking more suitable areas for their eggs to hatch [7]. Within the spring-to-autumn feeding season, such isolation may be characteristic of some lobster populations [58].

In this study, the movement process for lobsters consisted of long, directional steps (travelling) or short, non-directional steps (area-restricted movement), consistent with patterns seen in past research on lobsters and other species [3, 55, 74]. Shorter steps with lower directionality are recognized as more energetically expensive movements than longer steps with higher directionality and, based on previously characterised behaviours, these movements can be identified as searching or foraging and exploration, respectively [36, 55, 74]. Lobsters in this study exhibited most of their searching or foraging movements on *silt/mud with* ≤ *50% gravel*, corresponding to the strongest relative selection strength in the RSF analysis. iSSF analysis suggested that more exploratory movements occurred on *silt/mud*, *coarse sediments*, and *mixed sediments with a veneer of mud*. Because there was more area-restricted movement on the *silt/mud with* ≤ *50% gravel*, this substrate appeared to be more energetically costly to the lobsters, however, when compared to *silt/mud*, the area has potential for foraging that might also represent a net energy gain [49, 74]. *Silt/mud*, *coarse sediments*, and *mixed sediments with a veneer of mud* favoured movements that were the least energetically expensive and still provided potential foraging opportunities, however, they offer varying degrees of protection [26, 55, 74]. The preference shown for *coarse sediments* over *silt/mud* may result from the hardness of *coarse sediments* further reducing the energetic costs of moving on it. In addition to energy expenditures, opportunities for sheltering and food availability also differ among substrate types [31]. While many of these preferences were not found to have a significant effect on habitat selection, they do provide information that can be used to inform future hypotheses about lobster resource requirements and demonstrate the heterogeneity in the demands placed by lobsters on their habitats.

We observed wild lobster movements from a pseudo-random subset of the population, meaning that we were only able to observe lobsters that were captured, which might be a biassed sample due to capture vulnerability. Moreover, there were surely other lobsters sharing this same area that were not tagged, and the movements of these lobsters relative to untagged individuals may have had an influence that we could not observe [34]. RSF from telemetry data have yet to be applied as widely in the marine environment as they have in terrestrial settings (e.g., bears, [32]). To our knowledge this is the first application of an RSF to lobsters. However, our observations may alternatively reflect other aspects of lobster behaviour such as competition and territoriality that restricts individuals to specific boundaries. Future research will need to dive deeper into the mechanisms underlying lobster movement ecology to help understand the fundamental ecology and drivers of behavioral plasticity of these animals. In particular, experimental manipulations of habitat in mesocosms or manipulation of lobster densities would be illuminating to understand how abiotic and biotic constraints imposed in the wild influence measurements of habitat selection based on telemetry studies such as ours. Ranc et al. [44] conducted conceptually similar experiments on roe deer (*Capreolus capreolus*), demonstrating that deer returned to familiar sites within the home range even when resource availability within that range was manipulated. The relationship between internal (i.e. genetic, physiological) and external (i.e. competition, resource availability) mediators of movement ecology requires further study to understand how these dynamics can be expected to play out when these parameters are altered. Studies that integrate mark-recapture tagging of lobsters, such as Dunnington et al. [12] and O’Donnell et al. [42], with acoustic telemetry, similar to this work, would allow researchers to study more lobsters at a lower resolution while still gathering high resolution data from the acoustically tagged individuals. Mark-recapture studies also encourage increased engagement with fishers, as researchers rely on them to report captures of tagged lobster.

This study is limited in its sample size, as 72% of the tagged lobsters were removed from the iSSF analysis because the data they provided did not meet the requirements for the analysis. Some of these lobsters did not meet the requirements of iSSFs, which need high resolution data, while other individuals may have left the grid array or shed their external tags outside of the array (e.g., [4]). However, another study found that these lobsters appear to remain in this restricted area year-round even if they do not necessarily remain directly within the area covered by our positioning array [30]. Many insights into the movements of aquatic animals can be collected using acoustic telemetry, but the methodology can only concretely confirm the presence of an animal. Failure to detect a tagged animal may have multiple causes. It could indicate the animal is absent from an area, had died or was not moving into range of a receiver, or was present in the area but had lost its tag elsewhere, among other possibilities [23, 35, 46]. Indigenous knowledge helped fill some of the knowledge gaps resulting from limitations in study design and animal capture. For example, our partners at UINR shared their knowledge about lobster presence to inform receiver placement. Animal capture and release methods were also enhanced by our partner’s historical knowledge of seasonal movement patterns of lobster in the area. There are limitations inherent in all knowledge systems and methodologies, but the influence these limitations may have can be reduced when individuals with different knowledge systems work together. This sharing of knowledge was crucial to effectively defining the research questions and carrying out the study in a way that could address the needs of the community.

A key benefit of the project was the establishment of trust among partners as a foundation on which future necessary research work can be initiated. The area in which the Lake is situated is being subjected to rapid rises in temperature due to the effects of global climate change [11]. Given the geographic confines of the Lake, the species that are completing their entire life cycles within the system may face profound adaptation challenges. The negative impacts of climate change on the species important to the surrounding Mi’kmaq communities could have severe consequences for food security and the culture. The design of future research to understand the impacts of climate change in the face of these threats will depend on trusted relationships. Mi’kmaq knowledge will be especially important in identifying potential climate refuges for impacted species, and in co-designing experiments to confirm that these species can find and use the postulated refuges. In addition, the Mi’kmaq have discussed putting forward the Bras d’Or Lake as a potential future Indigenous Marine Protected Area. The co-design of the research and monitoring programs to define and maintain such an area will also be a future focus of research in the area, and future investigations will build on the trust developed in previous work.

The use of iSSFs can result in novel findings about animal movement and habitat selection processes, however, it can be challenging to conduct this type of analysis. Obtaining high resolution data about animals across a long period of time is very difficult, resulting typically in small sample sizes like those in this study. Simply establishing positions of lobsters at the bottom of the ocean is inherently a difficult task, and despite the limitations of the methods used here, those methods offer a framework for future studies. Studies using habitat selection models are likely to continue to improve and provide insights into selection processes on a seasonal scale, as certain substrates may be selected for during specific times of year, for example, as a result of prey items being predictably found in the area only at certain times, or due to seasonal migrations. This type of information would be valuable to managers in developing management plans that are adaptive and based on the local area.

Having a variety of substrates accessible to lobsters can allow individuals to exhibit diverse behaviours, forage for a variety of food, and remain close to their shelter [49]. However, availability of substrates may alter the extent to which behaviours are exhibited on substrates less suited to the behaviour [67]. The availability of substrates also influences distribution, because lobsters require their own shelters and space, or else they can be displaced through territoriality or possibly cannibalism [16, 27, 49, 67]. The influence of substrate availability on abundance arises from the different requirements of lobsters at different life stages [5, 9, 66, 69]. The adult lobsters used in this study are believed to have very little risk of predation, allowing them to exhibit more exploratory behaviours and likely reducing their association with substrates that provide protection from predation [49, 61]. However, the lobster population in the bay in this study is dependent upon local recruitment and juvenile lobsters require more shelter in their habitat than adults, owing to their higher risk of predation [10, 22, 49, 59]. It has been found that there is a strong interaction between the size of a lobster, its shelter requirements, and its foraging behaviours [31, 66] and foraging, in particular, presents a much higher risk for juveniles than adult lobsters [49].

Despite the small number of animals available for the habitat selection models, the RSF analysis suggested clear patterns indicating stronger selection of *silt/mud with* ≤ *50% gravel* than *coarse sediments* and *silt/mud* as the least selected compared to the reference class. The iSSF analysis did not suggest such strong associations, with three of the four lobsters that used *silt/mud* selecting it less than the reference class, though this was not a strong preference. The iSSF findings did indicate that four of the five lobsters had their shortest step lengths on the reference substrate. This is the first application of iSSF analysis to benthic marine animals using positions from acoustic telemetry, an avenue that merits further development to overcome limitations of accessing these environments to make observations about animals.

Understanding how aquatic habitats are used by culturally and commercially valued species is critical to effective management. This study demonstrated how research can be conducted by connecting knowledge systems of two different Nations with shared priorities. As part of Apoqnmatulti’k, this work used a framework to join together individuals with different worldviews with the goal of conducting research in an equitable environment that nurtured co-learning, and the partnership is now entering its fifth year. This unique collaboration was built on honest communication and a willingness to sit in discomfort. Project partners worked together to openly discuss shared and conflicting values and knowledge, to acknowledge power dynamics between individuals, and remained committed to working together, because of the shared belief that science is strengthened when diverse knowledge systems are valued. It is crucial that science is co-produced by Nations who share the land, waters, and resources. Identification of shared knowledge gaps between communities can foster science that contributes to strong management decisions that support Indigenous self-determination and address the realities of local lobster movements and habitat use.

## Data Availability

All data files are currently available at https://members.oceantrack.org/OTN/project?ccode=BDLSPG. Code used in this analysis is available at https://github.com/slandovs/bdl-lobster/tree/main.
